# CAP2 contributes to Parkinson’s disease diagnosed by neutrophil extracellular trap-related immune activity

**DOI:** 10.3389/fimmu.2024.1377409

**Published:** 2024-05-23

**Authors:** Xiaohe Li, Meiling Luo, Hangrui Xu, Lei Jia, Yanan Liang, Qianxi Xu, Yonghui Wang

**Affiliations:** Rehabilitation Center, Qilu Hospital of Shandong University, Jinan, China

**Keywords:** Parkinson’s disease, neutrophil extracellular trap, CAP2, immune system dysfunction, clinical diagnostic models

## Abstract

**Introduction:**

Neutrophil extracellular traps (NETs) constitute a crucial element of the immune system, and dysfunction in immune responses is implicated in the susceptibility and progression of Parkinson's disease (PD). Nevertheless, the mechanism connecting PD and NETs remains unclear. This study aims to uncover potential NETs-related immune biomarkers and elucidate their role in PD pathogenesis.

**Methods:**

Through differential gene analysis of PD and NETs in GSE7621 datasets, we identified two PD subtypes and explored potential biological pathways. Subsequently, using ClusterWGCNA, we pinpointed pertinent genes and developed clinical diagnostic models. We then optimized the chosen model and evaluated its association with immune infiltration. Validation was conducted using the GSE20163 dataset. Screening the single-cell dataset GSE132758 revealed cell populations associated with the identified gene.

**Results:**

Our findings identified XGB as the optimal diagnostic model, with CAP2 identified as a pivotal gene. The risk model effectively predicted overall diagnosis rates, demonstrating a robust correlation between infiltrating immune cells and genes related to the XGB model.

**Discussion:**

In conclusions, we identified PD subtypes and diagnostic genes associated with NETs, highlighting CAP2 as a pivotal gene. These findings have significant implications for understanding potential molecular mechanisms and treatments for PD.

## 1 Introduction

Parkinson’s disease (PD), first described by James Parkinson in 1817 (1), stands among the most prevalent neurodegenerative disorders. In 2016, over six million individuals worldwide were afflicted by PD, and this figure is anticipated to rise with the aging global population (2). Manifesting primarily as motor symptoms such as bradykinesia, rigidity, and tremor, PD also involves significant non-motor impairments (3). Noteworthy pathological changes encompass the absence of synaptic nucleoprotein α-positive inclusion bodies within neurons and axons, coupled with the loss of dopaminergic neurons in the substantia nigra and other brain regions (4). Besides, the role of genetic factors in PD has been paid more and more attention, and in recent years, a number of PD-GWAS results have been published in the world, revealing more than 90 risk gene loci. Hence, understanding genetic susceptibility and risk factors sheds light on the implicated pathogenic pathways (3, 5). PD exhibits substantial clinical variability and diverse prognoses, suggesting potential subtypes (6). Recognizing the importance of subtype identification, the National Institutes of Health has designated it as a top clinical research priority in PD (7). Consequently, delineating distinct PD subcategories is pivotal for comprehending underlying disease mechanisms, predicting disease progression, and designing effective clinical trials (8). However, methods for subtype identification and individual prognosis prediction remain elusive.

Dysregulation of the immune system is considered a pivotal factor in both the susceptibility and progression of PD. Numerous studies on Parkinson’s patients have documented markers of inflammation and immune cell populations that may initiate or worsen neuroinflammation, perpetuating the neurodegenerative process (9, 10). Moreover, growing evidence supports the role of an altered immune environment in PD pathogenesis (11). This has led to the hypothesis that intricate gene-environment interactions, combined with immune system activity, contribute to the ‘perfect storm,’ facilitating the development and progression of PD (12, 13). In consistent with this, analyses of blood from people with PD showed increased neutrophils and decreased lymphocytes many years before diagnosis, thus associating a higher neutrophil-to-lymphocyte ratio (NLR) with PD risk (14). This innate and adaptive compartment imbalance appears to be disease relevant, because the NLR correlates with the severity of motor defects, especially for tremor-dominant PD, and with striatal PET dopaminergic signal (15). Another study, in which people with PD were divided according to cognitive performance, further showed that those with mild cognitive impairment had a higher level of lymphocytes (i.e., lower NLR) compared with those with normal cognition (16). Although little is known about the role of neutrophils in PD, it seems to play a significant role in PD (14).

Notably, neutrophil extracellular traps (NETs) have entered the field of attention as a new concept of immunization. NETs, reticular structures released by neutrophils, play a crucial role in the immune response to infections. During NETosis, neutrophils release DNA strands along with antimicrobial proteins into the extracellular space, forming NETs. These structures trap and neutralize pathogens, preventing their spread and aiding in destruction (17). NETs production is implicated in various inflammatory, cardiovascular, and chronic diseases, making NETs not only a pathogenic factor but also a potential diagnostic or prognostic biomarker (18). Different types of exDNA such cfDNA or NETs have been identified under various pathophysiological conditions (including hyperinflammation, tumor progression or neurodegeneration) in the brain and can contribute to disease onset and progression in various ways (19–21). A common denominator in the pathogenesis is the release of mtDNA and cfDNA, the latter being particularly available in NETs, whereby both parameters may serve as disease biomarker (22). At present, studies in neurodegenerative diseases, such as multiple spinal sclerosis (23) and Alzheimer’s disease (24) have been investigated in relation to NETs, as well as PD has been reported to demonstrate a link between mtDNA-induced inflammation and PD (25), while the mechanism of the association with NETs is still unknown.

In this study, we utilized PD-related datasets from the GEO database and curated NRGs from the literature to identify DE-NRGs through analysis. We employed the GSVA algorithm to assess alterations in NETs-related signaling pathways in PD patients. Using ClusterWGCNA and machine learning algorithms, we identified DE-NRGs strongly correlated with PD. Clinical diagnostic models were then constructed to facilitate PD diagnosis and treatment. The CIBERSORT algorithm was employed to quantify immune cell infiltration percentages in PD patients. Our objective is to redefine PD classification using NETs as a risk predictor, uncover risk-associated genes, and investigate their relationships with immune cell populations.

## 2 Result

### 2.1 Differential gene analysis and principal component analysis

**Figure 1:**
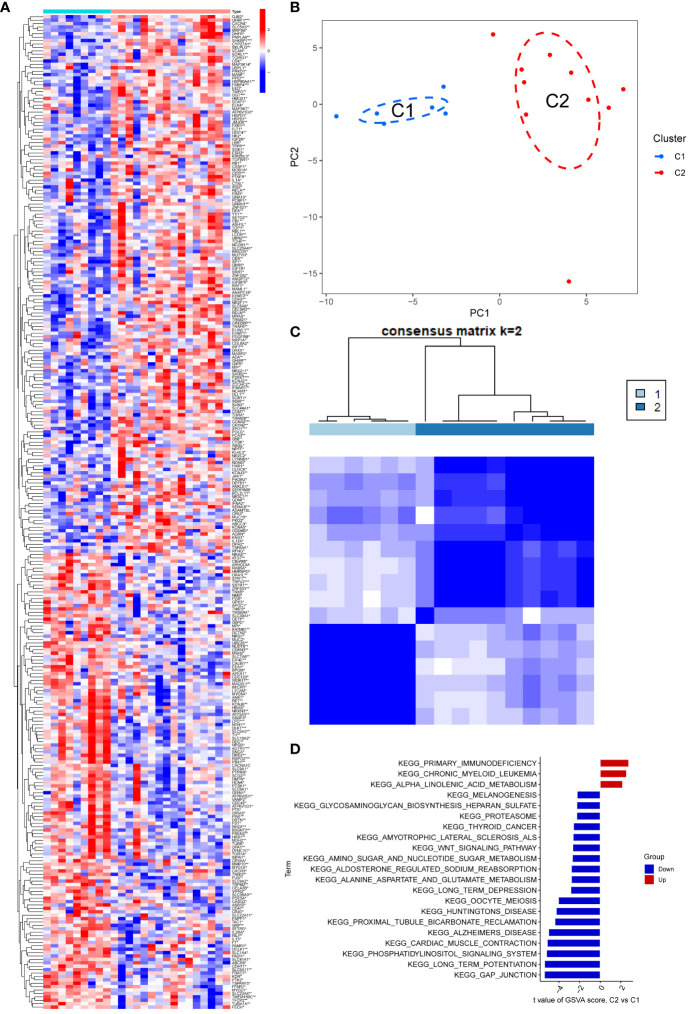
**(A)** The heat map showed that there were 292 differentially regulated genes, among which blue indicated down-regulated expression and red represented up-regulated expression; **(B, C)** PCA analysis re-divided the differentially differentiated genes into two isoforms, and when divided into two isoforms, K=2 had the best quality; **(D)** GSEA analysis pathway, red indicated expression enrichment in the pathway, and blue indicated that the biological pathway showed a state of inhibition.

In comparison to the normal group, the PD and NETs groups exhibited 292 differential genes (**Figure 1A**). To enhance the study of PD, we applied principal component analysis (PCA) to reclassify the differentially expressed genes, leading to the identification of two distinct subtypes, labeled as C1 and C2 (**Figures 1B, C**).

### 2.2 The biological pathways in which the two subtypes are involved

The potential biological subtypes of the two identified subtypes were further elucidated through Gene Set Variation Analysis (GSVA). Notably, C2 showed significant enrichment in primary immunodeficiency, chronic granulocytic leukaemia and α-linolenic acid metabolism compared to C1, especially in the case of primary immunodeficiency. Additionally, pathways related to gap junction, long-term potentiation, phosphatidylinositol signaling system, myocardial contraction, Alzheimer’s disease, proximal tubule bicarbonate recovery, and amyotrophic lateral sclerosis exhibited decreased expression in C2. In particular, gap junction and long-term potentiation showed prominent suppression, compared to a slight decline in melanogenesis and Biosynthesis of heparan sulfate aminoglycans (**Figure 1D**).

### 2.3 ClusterWGCNA

**Figure 2:**
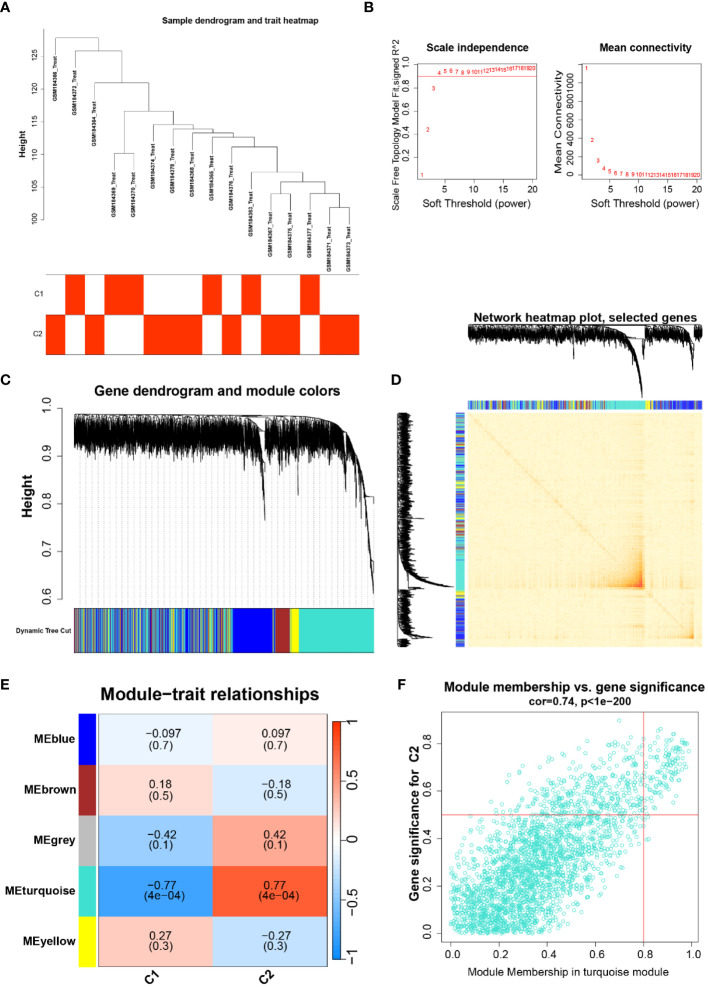
**(A)** Dendrogram and trait heat map of the sample, classification of the sample and its expression in the two subtypes of C1 and C2; **(B)** Soft thresholds for ClusterWGCNA enrichment analysis; **(C, D)** Gene dendrogram and gene co-expression network diagram; **(E)** Heatmap of the relationship between different traits and modules; **(F)** Gene significance in the emerald module, cor=0.74, P<1e-200.

To probe the highly correlated genes among the overlapping anoikis-related genes, we performed ClusterWGCNA to identify highly correlated gene modules (**Figures 2A, B**). Three gene modules were identified based on the gene tree: blue module, brown module, grey module, turquoise module, and yellow module (**Figures 2C, D**). A heat map showing the correlation between different modules in which there is a strong correlation between the occurrence of Parkinson’s and the turquoise module (coefficient 0.77, p-value 4E-04, **Figure 2E**). In addition, gene saliency (GS, i.e., correlation between genes and clinical traits) and module members (MM, i.e., correlation between genes and modules) in the turquoise module were highly correlated, indicating that the genes in this module had the most significant correlation with Parkinson’s (**Figure 2F**). Finally, we extracted 127 genes from the turquoise module for further analysis.

### 2.4 Construction of Parkinson’s related clinical diagnostic models

**Figure 3:**
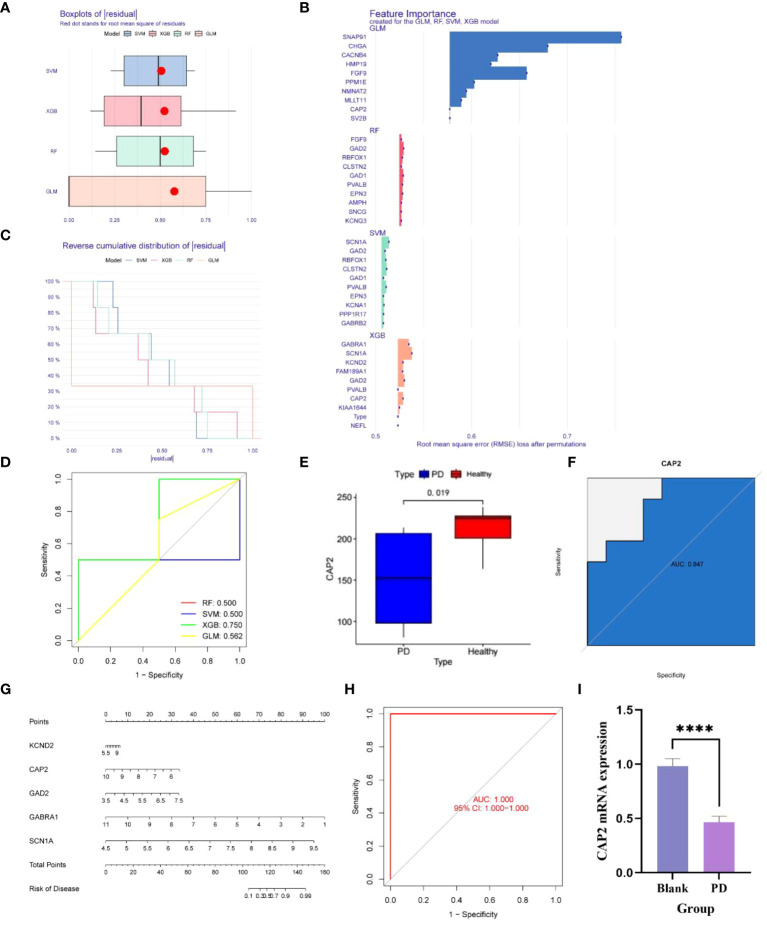
**(A, B)** The box plot shows that the differential genes are divided into four clinical diagnostic models - SVM, XGB, RF and GLM, where the red dot represents the root mean square of the residuals, and the smaller the residual value, the higher the diagnostic correctness; **(C, D)** The functional importance analysis of related genes in four clinical diagnostic models, SVM, XGB, RF and GLM, combined with the RCO curve, showed that the PF coefficient was 0.5, the SVM coefficient was 0.5, the XGB coefficient was 0.75, and the GLM coefficient was 0. 562; **(E)** The difference of the key gene CAP2 in the XGB model in the PD and normal groups was 0.019; **(F)** The survival curve shows that the AUC of CAP2 in the validation set is 0.847; **(G)** Risk Rating Scale: When the total score is less than 100, the risk of developing the disease is less than 0,1, and when the total score is between 100-150, the risk of developing the disease is 01-0.99, when the total score is greater than 160, the risk of disease is greater than 0.99; **(H)** ;The survival curve shows that the AUC of XGB is 1; **(I)** CAP2 mRNA expression between blank group and PD group (n=4, ****P<0.0001, t=11.78, df=6).

Utilizing the aforementioned 127 genes, LASSO, RF and SVM-RFE calculations were conducted, leading to the construction of four clinical diagnostic models—SVM, XGB, RF, and GLM. Higher accuracy in diagnosis was associated with lower residual values. Consequently, XGB and GLM demonstrated superior accuracy, while SVM and RF accuracy were comparatively lower (**Figures 3A, B**). The high-quality clinical diagnostic models were further refined, and the functional importance of related genes was analyzed using the SVM, XGB, RF, and GLM models. Combining this analysis with Receiver Operating Characteristic (ROC) curves—0.5, 0.75, 0.5, and 0.56, respectively—the XGB model was ultimately selected as the clinical diagnostic model (coefficient 0.75, **Figures 3C, D**). Subsequently, the XGB model was validated using the GSE20163 dataset ROC curve, achieving an AUC of 1, indicating robust performance for auxiliary diagnosis (**Figure 3H**). High-risk genes in the XGB model, including CAP2, GABRA1, GAD2, KCND2, and SCN1A, were identified. In the healthy and Parkinson’s groups, the expression of CAP2 gene in high-risk patients showed a significant down-regulation compared with low-risk patients (**Figures 3E, F**). q-PCR results confirmed that mRNA CAP2 expression in the Parkinson’s group was lower than that in the healthy group, aligning with the earlier analysis (**Figure 3I**). The nomogram illustrated that risk scores played a key role in predicting the total risk of CAP2, GABRA1, GAD2, KCND2, and SCN1A (**Figure 3G**). In conclusion, a NETs-related diagnostic risk model was successfully established, demonstrating robust performance in predicting the overall diagnosis of PD.

### 2.5 Enrichment analysis of GSEA

**Figure 4:**
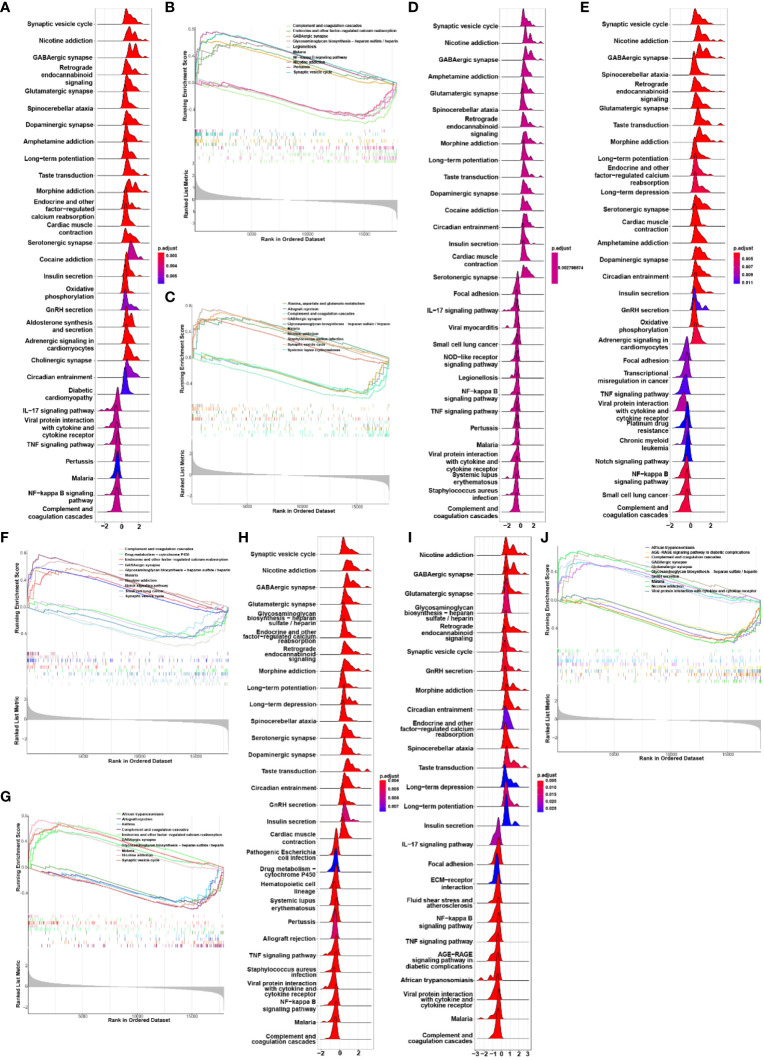
**(A, B)** GSEA analysis of biological signaling pathways associated with CAP2; **(C, D)** GSEA analysis of biosignaling pathways associated with GABRA1; **(E, F)** GSEA analysis of GAD2-related biological signaling pathways; **(G, H)** GSEA analysis of KCND2-related biological signaling pathways; **(I, J)** GSEA analysis of signaling pathways associated with SCN1A.

We conducted Gene Set Enrichment Analysis (GSEA) to evaluate the signaling pathways associated with the characteristic genes. CAP2 exhibited a positive correlation with endocrine and other factor-regulated calcium reabsorption, GABAergic synapse, glycosaminoglycan biosynthesis, nicotine addiction, and synaptic vesicle cycle. Conversely, it showed a negative association with complement and coagulation cascades, legionellosis, malaria, NF-kappa B signaling pathway, and pertussis (**Figures 4A, B**). Additionally, GABRA1 demonstrated associations with alanine, aspartate and glutamate metabolism, glycosaminoglycan biosynthesis, and GABAergic synapse (**Figures 4C, D**). GAD2 was linked to endocrine and other factors, glycosaminoglycan biosynthesis, and drug metabolism (**Figures 4E, F**). KCND2 participated in signaling pathways related to endocrine and other factor-regulated calcium reabsorption, nicotine addiction, and synaptic vesicle cycle (**Figures 4G, H**). SCN1A was involved in African trypanosomiasis, AGE-RAGE signaling pathway in diabetic complications, and complement and coagulation cascades (**Figures 4I, J**).

### 2.6 Immunoinfiltrate-related analysis

**Figure 5:**
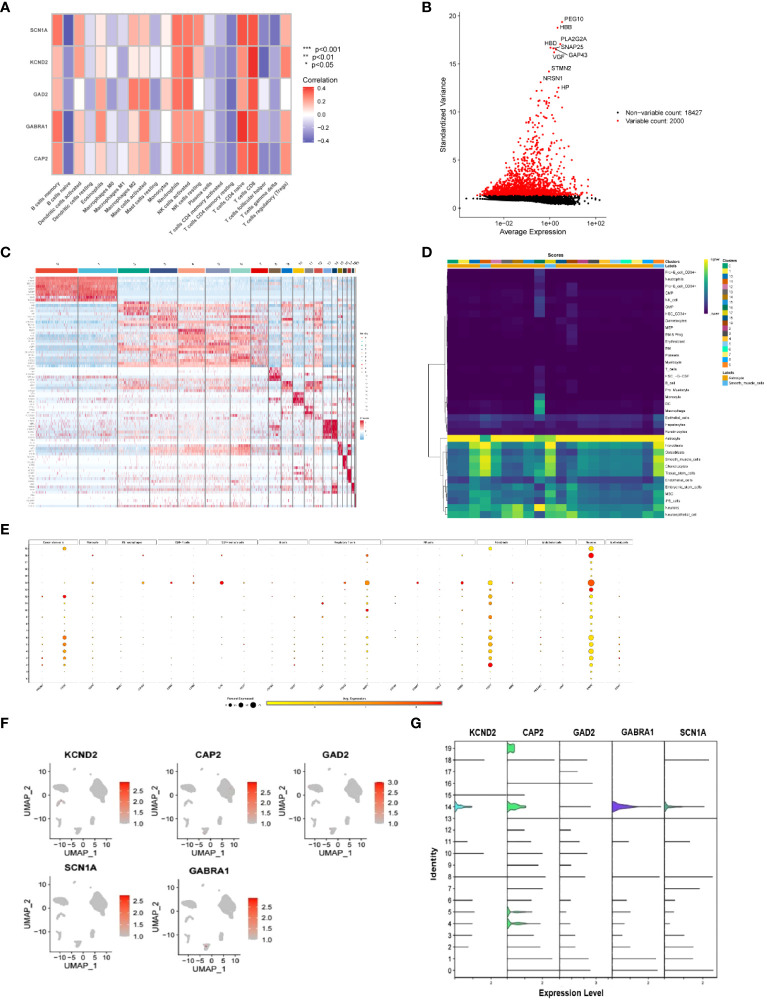
**(A)** Correlation of immune-infiltrating cells with key genes in the XBG clinical diagnostic model, red indicates active expression in immune cells in this gene, and blue indicates decreased expression in this cell; **(B)** Single-cell datasets were analyzed for standard variance; **(C)** Heat map of the genes associated with the genes of the 20 cells identified in this single-cell dataset, with red indicating upregulation and blue indicating downregulation; **(D)** The expression of the 20 identified cells in different types of cells, with high expression in yellow and low expression in blue; **(E)** Expression levels of cellular markers in different cell types of cells identified for 20 types of cells, with larger gardens indicating higher percentages, red indicating high expression, and yellow indicating low expression; **(F, G)** Expression of key genes in cells in the XBG clinical diagnostic model. .

To further investigate the correlation between infiltrating immune cells and the XGB clinical diagnostic models, we observed a particularly strong positive correlation between CAP2 and T cells CD8. Following this, T cells CD4 naïve, neutrophils, NK cells resting/activated, and B cells memory demonstrated involvement in the promotion of CAP2. Additionally, T cells CD8 were implicated in the activation of GABRA1, GAD2, KCND2, and SCN1A, with GABRA1 notably associated with T cells CD4 naïve. Furthermore, B cells naïve were identified as having a suppressive effect on CAP2, GABRA1, GAD2, KCND2, and SCN1 (**Figure 5A**).

### 2.7 Single-cell dataset validation

The cellular distribution of CAP2, GABRA1, GAD2, KCND2, and SCN1A, along with the involved cell types, was confirmed using single-cell data. The data revealed a total of 20 cell types, and the heat map displayed the highly expressed genes for each type. Notably, CD34, IL7R, NRP1, GZMB, FGF7, and ENO2 exhibited significant expression in cancer stem cells, CD4^+^ memory cells, regulatory T cells, NK cells, fibroblasts, and neurons, respectively (**Figures 5B–G**).

## 3 Discussion

In this investigation, we centered our attention on the influence of NETs-related genes on PD progression. A clinical diagnostic model was devised, enabling an exploration of the correlation between infiltrating immune cells, prognostic genes, and risk scores. To refine PD subtypes for improved treatment planning, we leveraged the latest immunological discovery, NETs, to reclassify PD into two subtypes, namely C1 and C2. Subsequently, key genes linked to C1 and C2 were identified using ClusterWGCNA, leading to the creation of four clinical diagnostic models. Among these models, the XGB clinical model exhibited superior diagnostic value, with the CAP2 gene showing significant differences. Biological processes and immune cell infiltration within the subtypes were dissected using GSVA and GSEA, unveiling GABAergic synaptic activation, spinocerebellar ataxia, and calcium reabsorption. Lastly, we explored the correlation between infiltrating immune cells and prognostic genes, discovering significant correlations between risk scores and most immune cells, as well as strong correlations between prognostic genes and most immune cells. In conclusions, our findings propose that NETs-associated genes offer insights into the prognostic significance and potential for immunotherapy in PD.

GSVA analyses unveiled that the new isoforms were predominantly enriched in biological pathways related to gap junction ns, long-term potentiation, and myocardial contraction. This aligns with a prior report on multimodal imaging methods for PD, which identified dysfunction in the gut, heart, brainstem (locus coeruleus), and nigral projections (26). The GSVA analysis results indicated that the new subtypes were largely enriched in pathways associated with gap junctions, long-term potentiation, and myocardial contraction, suggesting a connection to both motor and cardiac symptoms of PD. Previous subtypes commonly exhibited debilitating motor and non-motor symptoms associated with adaptive changes at the cellular and synaptic levels within neural circuits (27). Exercise-enhanced neuroplasticity has been recognized for its potential to target motor and cognitive circuits in PD (28, 29). Moreover, the reclassification of PD revealed related enrichment manifestations in Alzheimer’s disease and amyotrophic lateral sclerosis, highlighting a common feature of dyssynaptic function in various brain diseases, including the mentioned neurodegenerative diseases (30). However, past research has lacked an exploration of associations between disease types, with few studies investigating potential links and mechanisms.

The genomics era has brought rapid advancements in understanding the genetic causes and risk variants of PD (31). Validating clinically defined disease subtypes requires objective biological measures or biomarkers indicating differences in underlying disease mechanisms or pathology (32, 33). Our reanalysis based on the PD subtypes described above disclosed inhibition of the phosphatidylinositol signaling pathway in PD patients, aligning with previous studies demonstrating genetic risk factors for PD with lipid-related functions (34). Furthermore, we assessed the signaling pathways of genes associated with the XGB clinical diagnostic model through GSEA. Positive circuits in endocrine and other factor-regulated calcium uptake included calcium uptake, GABAergic synapses, nicotine addiction, and synaptic vesicle cycling. Conversely, negative regulation was observed in complement and coagulation cascade responses, malaria, and NF-κB signaling pathways. These findings resonate with previous studies emphasizing abnormal α-synuclein aggregation, mitochondrial dysfunction, lysosomal or vesicular transport issues, synaptic transport problems, and neuroinflammation as contributors to the pathophysiological changes in PD (4). Established pathways include the link between α-synuclein and lysosomal acid GCase forming a positive feedback loop, potentially leading to a self-propagating disease. Additionally, a pathological cascade commencing with mitochondrial oxidant stress, resulting in oxidized dopamine accumulation, reduced lysosomal acid GCase activity, and subsequent α-synuclein accumulation (35–37).

Cyclase-associated proteins (CAPs) are evolutionarily conserved actin-binding proteins crucial for regulating actin dynamics, governing the spatiotemporal assembly and disassembly of actin filaments (F-actin) (38–40). Mammals have two family members with different expression patterns (CAP1 and CAP2). Unlike most other tissues, both CAPs are expressed in the brain and present in hippocampal neurons. Among them, CAP2 is the main family member in striated muscle (41). It interacts with the actin depolymerization protein Cofilin1, a key regulator of synaptic actin dynamics, spine morphology, synaptic plasticity, brain function and behavior (27, 29, 34, 42–44). In fact, CAP2 can control dendritic spine morphology and synaptic plasticity (45). In this study, we found that the expression of CAP2 was upregulated in the low-risk group compared to the high-risk group. In addition, the results of the validation cohort showed that the expression of CAP2 in the Parkinson’s sample was downregulated compared to the normal sample. When the expression of CAP2 is reduced in PD, synaptic plasticity, brain function and motor function will be affected, and related symptoms such as cognitive dysfunction and tremor will appear. Therefore, combined with our research, it can be inferred that CAP2 may affect Parkinson’s motor function limitation by affecting the connection between the long-term potentials and gaps of synapses, resulting in a series of related motor function changes such as bradykinesia, rigidity, and tremor.

Dopamine is a pivotal factor in PD development and serves as a key immunomodulator. Various immune cells express dopamine receptors and dopaminergic proteins, participating in dopamine ingestion, production, storage, and release (13). Besides, dopamine-induced extracellular traps (ETs) are functional (46). There are two main functional regions of NETs present: the generation of oversized NETs scaffolds (consisting of whole decompressed nucDNA, histones, and various antimicrobial proteins and enzymes) used to trap and kill microorganisms in the initial immune response when neutrophils are stimulated; and the other, where activated platelets also act as an inducer of NETosis by providing adhesive interactions with neutrophils, which ultimately results in the immediate formation of cellular aggregates, from which the NETs are released to stimulate prothrombotic functions (22). Meanwhile, not only neutrophils but also mast cells, eosinophils, basophils, macrophages and also microglial cells as the resident immune cell of the CNS have been described to release nucDNA-containing ETs in response to various stimuli (47, 48). When neuronal function is compromised in PD, neuronal cell death is preceded by activation of microglia (49). The study of microglia has been shown in multiple occasions in several PD models (50, 51).

Furthermore, strong dysregulation of peripheral monocytes in PD patients, including subpopulation shifts and impaired secretion of inflammatory molecules in response to stimulation. Previous studies on Parkinson’s brains and peripheral T cell subsets revealed CD3 T cell infiltration in the brains of Parkinson’s patients (52). Moreover, CD4 and CD8 T cells are found in substantia nigra dense bodies in Parkinson’s patients, exhibiting higher levels than in the normal group (53). CD4^+^ and CD8^+^ lymphocytes were found in the blood and cerebrospinal fluid of idiopathic patients, indicating peripheral activation of lymphocytes in addition to elevated levels of IL-1 β, TNF-α and IL-2 (54). Our investigation identified a strong positive correlation between CAP2 and T cells CD8, as well as T cells CD4 naïve. Similarly, altered peripheral CD4^+^, CD8^+^, CD3^+^, and CD4^+^/CD8^+^ levels have been reported in cognitively impaired PD patients (55). Parkinson’s patients also displayed increased HLA-DR T cells and CD45RO memory T cells, with a simultaneous decrease in naïve CD4 T cells compared to healthy controls (56, 57). In accordance with it, Parkinson’s patients with more severe trajectories of cognitive deterioration exhibited higher levels of circulating lymphocytes (16). Additionally, our study revealed that CAP2 positively regulates neutrophils, natural killer (NK) cells, and B cells memory. Notably, NK cells which are responsible for clearing α-synuclein aggregates, the primary component of Lewy bodies, has been reported to play a crucial role in PD. Systemic depletion of NK cells in mouse models of α-synucleinopathy leads to neuropathological deterioration, highlighting their relevance in PD. However, the exact role of NK cells in PD remains unclear. Single-cell dataset analysis further indicated that cancer stem cells, CD4^+^ memory cells, regulatory T cells, NK cells, fibroblasts, and neurons participate in the regulation of related genes, with GZMB serving as a marker for NK cells.

Overall, we identified CAP2 as a key gene via the establishment of two NET-associated PD subtypes, C1 and C2, and a diagnostic model for XGB. These findings have important implications for understanding potential molecular mechanisms and therapeutic approaches for degenerative brain disorders.

While our study represents a pioneering effort in identifying subgroups and prognostic genes related to NETs in PD, several limitations should be acknowledged. Firstly, the total cohort size and available sequencing data are limited. Secondly, this study lacks extensive basic experiments to validate the expression of prognostic genes in Parkinson’s cell lines and to elucidate the involvement of associated immune cells, necessitating further research.

## 4 Materials and methods

### 4.1 Data collection and processing

We downloaded PD expression profile data (GSE7621) (PD sample=16, normal sample=9, substantia nigra tissue), validation group expression profile data (GSE20163) (PD sample=8, normal sample=9, substantia nigra tissue), and single-cell expression profile data (GSE132758, perivascular-like cells in stem cell-derived grafts) from NCBI GEO.

### 4.2 Differential gene and principal component analysis

We used the edge R package to analyze the difference between the two sets of data (the threshold was set to log2|FC=>1,p < 0.05). In order to directly display the deg between the PD sample and the normal sample, a heat map is drawn using the p heatmap package. We use Principal Component (PC) Analysis (PCA) to identify the set of signals that vary in concert (called covariances) across many NETs. This method generates thousands of PCs, each capturing different patterns of covariance across many NETs. We then used penalized regression to exclude PCs that were not relevant to PD (including those driven primarily by noise), obtaining a PD with only 2 PCs.

### 4.3 Subclusters analysis with two NETs -related genes

The “ConsensusClusterPlus” R package (58) and the mRNA expression of two Unsupervised hierarchical cluster analysis of PD samples was performed using NETs-related genes as input information. When we looked at the subclusters using a PCA plot, we could see the geometrical distance between them. GSVA (59) was applied to clearly state the functional distinctions between the subclusters found via previous cluster analysis.

### 4.4 Enrichment analysis of GSEA

Gene set enrichment analysis (GSEA) was performed on GSE20163 using the “GSEA” R software package to study the relevant pathways of candidate diagnostic genes, and the reference gene set was KEGG. The number of random sample permutations was set to 1000 and p<0.05 was considered significant enrichment.

### 4.5 Enrichment analysis of ClusterWGCNA

The gene co-expression network of PD and NETs in the dataset was GSE7621 constructed using the WGCNA of the expression profile of “WGCNA” in the R package. The network construction process mainly consists of the following steps: 1. Define the similarity matrix. 2. Select the weight factor β = 12 to convert the similarity matrix to an adjacency matrix. 3. Convert the adjacency matrix to a topological overlap matrix (TOM). 4. The dissTOM is stratified based on Tom clustering to obtain a hierarchical clustering tree. 5. Use the dynamic tree cutting method to identify modules from the hierarchical clustering tree. 6. Calculate the module characteristic genes (MEs) for each module, where MEs represent the overall expression level of the module. The Pearson correlation coefficient between the MEs of each module has been calculated, and the 1-Pearson correlation coefficient is defined as the average distance between the MEs of each module. The average linkage hierarchical clustering method has been used to cluster the MEs of all modules, and the minimum value (genome) was set to 100. Modules with high similarity are combined to obtain a co-expression network.

### 4.6 Machine learning algorithm for candidate genes

After identifying the DEGs, we performed three machine algorithms, namely Minimum Absolute Shrinkage and Selection Operator (LASSO) logistic regression, random forest (RF), and support vector machine recursive feature elimination (SVM-RFE) to screen PD candidate genes using “glmnet”, “randomforest”, and “e1071” packages, each located in the R software. We then performed further analysis using genes from LASSO, RF and SVM-RFE algorithms. Expression of candidate genes was first verified in GSE7621 datasets, and both sides of P < 0.05 were considered statistically significant. Ultimately, the area under the receiver operating characteristic (ROC) curve (AUC) was calculated to assess the accuracy of the selected gene in diagnosing patients with PD.

### 4.7 Immunoinfiltrate-related analysis

CIBERSORT performed immunoinfiltration analysis of the difference in GSE20163 to observe the difference between PD and normal tissue immune cell infiltration, and visualized it through bar graphs, correlation plots, thermal images and violin plots. Correlation analysis of hub genes with immune-infiltrating cells. Taking the median gene expression as the boundary, the five hub genes were divided into high and low groups, and the box plot was used to visually observe whether there was any difference in the expression of immune-infiltrating cells between the high and low groups.

### 4.8 Single-cell dataset validation

To further explore the relationship between immune cells and diagnostic model genes, we performed single-cell data analysis on GSE132758 datasets and calculated mitochondrial gene expression using the Percent Feature Set function of the Seurat software package (60). Cells with >25% UMI in the mitochondrial genome are removed by quality control. The integration matrix is then scaled and the first 30 dimensions of principal component analysis (PCA) are used for t-distribution random neighborhood embedding (t-SNE) visualization. We apply the same scaling, dimensionality reduction, and clustering processes to specific datasets for sub-clusters. We used the Wilcoxon rank-sum test to identify significantly differentially expressed genes (DEGs) in each cluster by comparing other clusters. Single R and primary marker genes for the identification of cell types (61). DEGs significantly upregulated by Top100 were imported into the STRING website (http://string-db.org/) for further analysis to screen for hub differential genes in Cytoscape software. In general, the genes with the most connections are the most important genes in the module.

### 4.9 q-PCR

Total mRNA was extracted from the Parkinson’s cell lines LUHME. After purification, the RNA is eluted with enzyme-free water and its concentration and purty are determined. Reverse transcription and amplification reactions were performed using reverse transcription kits and fluorochrome kits. The primers are designed based on sequences found in GenBank. The sequence of primer nucleotides used in this study is shown in **Table 1**. The q-PCR system is 20 μL and includes 7 μL of DEPC water, 10 μL of TB Green ^®^ Premix Ex TaqTM II, 0.4 μL of PCR forward primer (10 μM), 0.4 μL of PCR reverse primer (10 μM), and 1 μL of cDNA. This should be repeated for each well. Reverse transcription reaction conditions: 37°C, 15 min, 85°C, 5 sec, amplification conditions: 95°C, 30 sec, 95°C, 30 sec: 95°C, 30 sec, 95°C, 5 sec, 60°C, 30 sec, 40 cycles. Using GAPDH as an internal control, the CT values of each group were counted, and the data were analyzed with 2-ΔΔ CT (Livak method).

**Table 1:** The sequence of primer nucleotides used in this study.

	FORWARD	REVERSE
CAP2	TGTCAGCCGCCTGGAGTCG	TGGATGCTACAGGACCCTCGTG
GAPDH	CTGGAGAAACCTGCCAAGTATG	GGTGGAAGAATGGGAGTTGCT

### 4.10 Data analysis

All analyses were performed using R software (version 3.6.2), and a P value of less than 0.05 was considered statistically significant. The qRT-PCR data obtained are expressed as mean ± standard error (SEM). Student’s t-test or one-way ANOVA was used to compare differences between groups. GP software was used for statistical analysis. P < 0.05 was statistically significant.

## Data availability statement

Publicly available datasets were analyzed in this study. This data can be found here: https://www.ncbi.nlm.nih.gov/geo/query/acc.cgi?acc=GSE7621, https://www.ncbi.nlm.nih.gov/geo/query/acc.cgi?acc=GSE20163, https://www.ncbi.nlm.nih.gov/geo/query/acc.cgi?acc=GSE132758.

## Ethics statement

Ethical approval was not required for the studies on humans in accordance with the local legislation and institutional requirements because only commercially available established cell lines were used.

## Author contributions

XL: Data curation, Methodology, Software, Validation, Writing – original draft, Writing – review & editing. ML: Formal analysis, Methodology, Validation, Writing – original draft, Writing – review & editing. HX: Data curation, Investigation, Supervision, Validation, Writing – review & editing. LJ: Funding acquisition, Investigation, Supervision, Writing – review & editing. YL: Supervision, Validation, Writing – review & editing. QX: Supervision, Validation, Writing – review & editing. YW: Conceptualization, Funding acquisition, Project administration, Resources, Supervision, Writing – review & editing.

## Supplementary material

The expression of other genes in XGB in high-risk patients showed a significant down-regulation compared with low-risk patients (**Supplementary Figure S1**).
