# Supplementary figures and images for: CAP2 contributes to Parkinson’s disease diagnosed by neutrophil extracellular trap-related immune activity
Source: Front Immunol. 2024 May 23;15:1377409. doi: 10.3389/fimmu.2024.1377409 (PMC11153744; doi:10.3389/fimmu.2024.1377409)

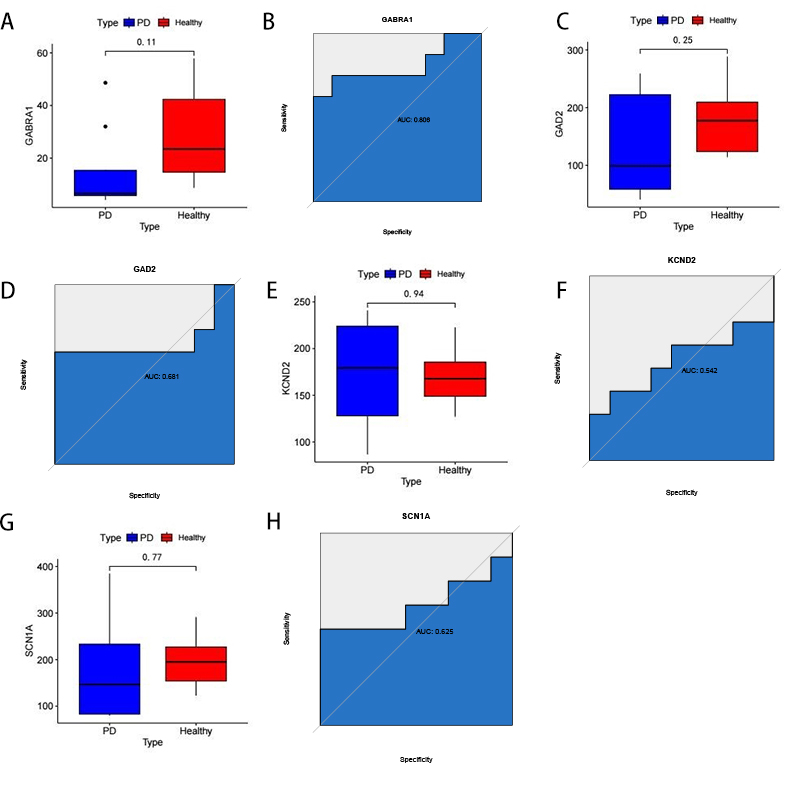

Supplement: Supplementary file 1 [file Image_1.tif]
